# Dexamethasone Chemotherapy Does Not Disrupt Orexin Signaling

**DOI:** 10.1371/journal.pone.0168731

**Published:** 2016-12-20

**Authors:** David E. Kram, Stephanie M. Krasnow, Peter R. Levasseur, Xinxia Zhu, Linda C. Stork, Daniel L. Marks

**Affiliations:** 1 Division of Pediatric Hematology-Oncology, Doernbecher Children’s Hospital/Oregon Health & Science University, Portland, Oregon, United States of America; 2 Papé Family Pediatric Research Institute, Oregon Health & Science University, Portland, Oregon, United States of America; Universite Clermont Auvergne, FRANCE

## Abstract

**Background:**

Steroid-induced sleep disturbance is a common and highly distressing morbidity for children receiving steroid chemotherapy for the treatment of pediatric acute lymphoblastic leukemia (ALL). Sleep disturbance can negatively impact overall quality of life, neurodevelopment, memory consolidation, and wound healing. Hypothalamic orexin neurons are influential wake-promoting neurons, and disturbances in orexin signaling leads to abnormal sleep behavior. A new class of drug, the orexin receptor antagonists, could be an intriguing option for sleep disorders caused by increased orexinergic output. Our aim was to examine the impact of ALL treatment doses of corticosteroids on the orexin system in rodents and in children undergoing treatment for childhood ALL.

**Methods:**

We administered repeated injections of dexamethasone to rodents and measured responsive orexin neural activity compared to controls. In children with newly diagnosed standard risk B-cell ALL receiving dexamethasone therapy per Children’s Oncology Group (COG) induction therapy from 2014–2016, we collected pre- and during-steroids matched CSF samples and measured the impact of steroids on CSF orexin concentration.

**Results:**

In both rodents, all markers orexin signaling, including orexin neural output and orexin receptor expression, were preserved in the setting of dexamethasone. Additionally, we did not detect a difference in pre- and during-dexamethasone CSF orexin concentrations in children receiving dexamethasone.

**Conclusions:**

Our results demonstrate that rodent and human orexin physiology is largely preserved in the setting of high dose dexamethasone. The data obtained in our experimental model fail to demonstrate a causative role for disruption of the orexin pathway in steroid-induced sleep disturbance.

## Introduction

The long-term survival rate of greater than 90% for childhood acute lymphoblastic leukemia (ALL) has been achieved using multi-agent cytotoxic chemotherapy that employs a glucocorticoid backbone.[1] High-dose glucocorticoids, however, are also associated with many serious adverse events, including severe avascular necrosis, behavioral changes, increased fatigue, and sleep disturbances.[2, 3] Sleep disturbances can have profound influences on a child’s overall well-being while undergoing therapy for ALL.

Sleep is a highly complex, coordinated, and integrative neurologic process vital for its restorative value: it reduces fatigue, lethargy, mood disturbance, school absenteeism, and general poor productivity.[4] Sleep is also crucial for memory consolidation, immune functioning, wound healing, and overall quality of life.[5] Children with ALL and their parents rate disrupted sleep as one of the most distressing symptoms related to ALL maintenance therapy.[6] Survey studies show disturbed sleep patterns in children during dexamethasone treatment, including frequent nighttime awakenings, restless sleep, and increased daytime sleepiness.[6, 7] Actigraphy, a reliable tool for evaluating sleep patterns in humans, is used to corroborate these reported sleep disturbances in children with ALL during dexamethasone pulses.[7–9] However, despite decades using glucocorticoids for ALL and many other diseases, how these synthetic hormones cause sleep disturbance remains unclear.

Orexins are neuropeptides produced in the brain exclusively by a group of neurons located in the hypothalamus that have profound influence on arousal and sleep in humans and animals. By stimulating wake-active monoaminergic and cholinergic neurons in the hypothalamus and brain stem during the daytime, orexins play a key role in maintaining a long, consolidated awake period.[10, 11] Orexin neurons anatomically located in the perifornical area (PFA)-dorsomedial hypothalamic area (DMH) promote wakefulness, while those located in the lateral hypothalamus (LH) drive reward-seeking behavior.[12, 13] Loss of orexin signaling causes narcolepsy in humans and animals and highlights orexin’s pivotal role in regulating the awake-sleep switch.[10–15] Mice that lack orexin neurons, orexin neuropeptide, or the orexin receptors display symptoms of excessive sleepiness or complete cataplectic narcolepsy.[16–18] Furthermore, orexin receptor antagonist drugs are sleep promotors, used to treat insomnia in humans.[19] Conversely, when orexin is overexpressed, wakefulness and poor sleep is intensified.[20, 21]

Two lines of evidence suggest that orexin overexpression may mediate steroid-induced sleep disturbance. First, rodents with supra-physiologic levels of orexin exhibit many behaviors seen in humans receiving exogenous steroids, including hyperlocomotion, fragmented non-rapid eye movement (NREM) sleep frequently disturbed by short episode of wakefulness, reduction in rapid eye movement (REM) sleep, and increased awake time.[14, 15, 20–24] Second, orexin neurons play a role in regulating the hypothalamic-pituitary-adrenal (HPA) axis and the eventual production of cortisol.[25] Disruption of the normal diurnal rhythm of cortisol in cancer treatment has been linked to sleep problems.[26] High-dose corticosteroids greatly impact the functioning of the HPA axis and the rhythmicity of cortisol, and the finding that adrenalectomy decreases hypothalamic orexin expression suggests that the orexin system may be the mediator between HPA axis dysfunction and sleep disturbances.[27, 28]

Orexin receptor antagonists are a new class of drugs aimed at reducing orexinergic tone during the night to mitigate hyperarousal and improve sleep.[19] Having recently received US Federal Drug Administration (FDA) approval for the treatment of insomnia following phase 2 and 3 trials, orexin receptor antagonists would be an intriguing option for other sleep disorders caused by increased orexinergic tone.[29] In the present work, we sought to investigate the relationship between the orexin system and exogenous corticosteroid administration in mice, rats, and humans.

## Materials and Methods

### Animals

Male C57BL/6J mice (12 weeks) were purchased from Jackson Laboratories (Bar Harbor, ME). Male CRL:CD (SD) rats (101–125 g) were purchased from Charles River Laboratories (Wilmington, MA). Mice were used to measure the impact of dexamethasone on quantitative and anatomic orexin neuron gene expression. Rats were used to measure the effect of dexamethasone on quantitative orexin neuron gene expression, quantitative hypothalamic orexin protein concentration, and cerebral spinal fluid (CSF) concentrations of orexin. All animals were maintained in a pathogen-free room on a normal 12 h light/dark period with lights on from 06:00 to 18:00 at 22–24°C with *ad libitum* access to food (rodent diet 5001, Purina Mills) and water. Animals and leftover food were weighed daily during experiments. Animal experiments were conducted in accordance with the National Institutes of Health *Guide for the Care and Use of Laboratory Animals* and approved by the Oregon Health & Science University (OHSU) Department of Comparative Medicine Institutional Animal Use and Care Committee.

### Animal steroid administration

Dexamethasone Sodium Phosphate Injection (DEX) 4 mg/mL was obtained from the OHSU Pharmacy (Fresenius Kabi, NDC 63323-0165-01). Animals were administered DEX twice daily for 5 days at a concentration of 1.5 mg/kg in mice and 3.0 mg/kg in rats. This drug regimen reflects the steroid burst used in maintenance chemotherapy for children with standard risk (SR) ALL.[30] The body surface normalization method based on FDA recommendations was used to calculate the mouse and rat doses in mg/kg based on the human dose of DEX (6 mg/m^2^ divided BID for 5 days).[31] Vehicle-treated animals were injected with an equivalent volume of normal saline (vehicle) to DEX-treated animals. DEX or vehicle was administered via intraperitoneal (IP) injections between 06:00–07:00 and 17:00–18:00. Animals were returned to their home cages after injection.

### Animal whole brain and hypothalamus collection

On experiment days 1–5, the animals were injected IP with DEX or vehicle. On experiment day 5 between 16:00–18:00, animals were deeply anesthetized (mice with a ketamine-xylazine-acepromazine cocktail, rats with 4% isofluorane), decapitated, and whole brains removed. This time point coincides with the expected orexin nadir.[10] For mouse *in situ* hybridization, whole brains were frozen on dry ice and stored at -80°C until the time of assay. For mice and rat hypothalamus mRNA expression and for rat hypothalamus quantitative orexin protein analysis, hypothalamic blocks (including the hypothalamus, most diencephalic structures, and the prefrontal cortex) were excised from the whole brain as described previously.[32] Blocks were preserved in RNAlater solution (Ambion), stored at 4°C overnight, then frozen at -80°C without RNAlater solution until the time of assay.

### Measuring the impact of corticosteroids on the orexin system

Orexin neuronal activity can be inferred from measurements of prepro-orexin mRNA and orexin neuropeptide. In addition, the expression mRNA coding for orexin receptors in target neurons is also linked to rodent behavior.[33, 34] The *prepro-orexin* gene encodes for orexin neuropeptides, and activity of this gene can be measured by prepro-orexin mRNA. Orexin neuropeptides are detectable both locally in hypothalamic tissue and distally in CSF. Orexin receptor 1 (OX_1_R) mRNA and orexin receptor 2 (OX_2_R) are orexin receptors, both of which are important in the regulation of sleep and maintenance of arousal.[35] OX_1_R and OX_2_R double knockout mice and dogs exhibit similar narcoleptic behaviors and electroencephalographic phenotypes to *prepro-orexin* knockout animals.[16, 36] Therefore, we comprehensively examined the impact of corticosteroids on orexin activity by measuring orexin gene expression, orexin neurotransmitter production, and orexin receptor expression.

### RNA preparation and relative quantitative RT-PCR in mice and rats

Total RNA was extracted from brain tissue using QIAGEN RNeasy kits (QIAGEN, Inc., Valencia, CA) and as previously reported.[37] cDNA was synthesized for both, and RT-PCR reactions were run using revalidated TaqMan master mix and rat-specific primer-probe (Applied Biosystems). Relative levels of mice prepro-orexin mRNA, OX_1_R mRNA, and OX_2_R mRNA were measured.

### Hypothalamic Orexin-A protein preparation for RIA in rats

Orexin-A protein extraction from the hypothalamic tissue for RIA was performed according to the manufacturer’s instructions and as previously described (Phoenix Pharmaceuticals, Burlingame, CA).[38]

### *In situ* hybridization (ISH) histochemistry for orexin (Hcrt) mRNA in mice

Preparation of coronal brain sections and single ISH were performed as previously described.[32] Antisense ^33^P-labeled rat prepro-orexin (*Hcrt*) riboprobe with near complete homology to mice (corresponding to bases 18–420 of rat *Hcrt*; GenBank accession number NM_013179)(0.1 pmol/mL) was applied to slides. Blinded counts of the number of sliver grain clusters (corresponding to radiolabeled *Hcrt* mRNA) in each hypothalamic nucleus, differentiated by neurons located in the PFA-DMH versus LH (user defined), as well as the number of sliver grains in each cell, were made using Grains 2.0.b software (University of Washington, Seattle, WA).

### Rat CSF sample serial collections

CSF was collected with a novel method, described and shown in S1 Fig.

### Patient subjects

Eligible subjects included all newly diagnosed patients between 2014–2016 at our institution with SR B-cell ALL treated according to Children’s Oncology Group (COG) induction therapy with 28 days of dexamethasone (6 mg/m2/day)[30]. Involvement by eligible patients in this study was not based on a standard consenting process, but rather based on an opt-out procedure and was approved by the OHSU Institutional Review Board. Compliant with the “Policy for Accessing Tissue Specimens or Information at OHSU for Anonymous or Coded Genetic Research” that allows de-identified samples obtained from standard of care procedures to be used for future research provided individuals do not opt out, we provided a study information sheet to all participating patients/parents/guardians that included information on this study as well as details about the opt out process for this research. Patient samples were included in this study if the patients/parents/guardians did not opt out.

### Human CSF sample collections

One milliliter of CSF was collected at the time of standard-of-care therapeutic lumbar punctures (LPs) on Days 0 and 8 of induction chemotherapy (*n* = 22). Day 0 of induction represented the baseline orexin level; Day 8 represented 7 days into DEX treatment. While CSF samples were also collected at later time points, Days 0 and 8 were tested in this study in order to simulate the 5-day burst of steroid treatment during maintenance therapy of ALL. Specific time of day of CSF collection was not standardized. Samples were stored in 500 μL aliquots at -80°C until the time of assay.

### Rat CSF and hypothalamus, and human CSF orexin-A levels by orexin-A radioimmunoassay (RIA)

Rat and human CSF were prepared and processed using the same technique. Frozen samples were thawed on ice. Duplicates of 25 μL of CSF were used for each RIA. Lyophilized hypothalamic protein extract was re-dissolved in 250 μL of RIA buffer. Orexin-A levels were determined in duplicate for all samples using a commercial radioimmunoassay (RIA) kit (Phoenix Pharmaceuticals, Burlingame, CA) according to manufacturer’s instructions. The detection limit was 10 pg/mL for all samples and intra-assay variability was <10% for rat hypothalamus, rat CSF, and human CSF.

### Statistical analysis

Data were graphed and analyzed using GraphPad Prism 5. All comparisons were made between two groups at a single time point and were performed using two-tailed Student’s t test.

For the human CSF study with 25 samples, this study has been powered to have an 80% chance of detecting a 20% increase in mean of CSF orexin concentration from baseline to Induction Day 8. All differences between groups were considered significant when p < 0.05.

## Results

### Orexin signaling appears to be preserved following dexamethasone administration

**Fig 1:**
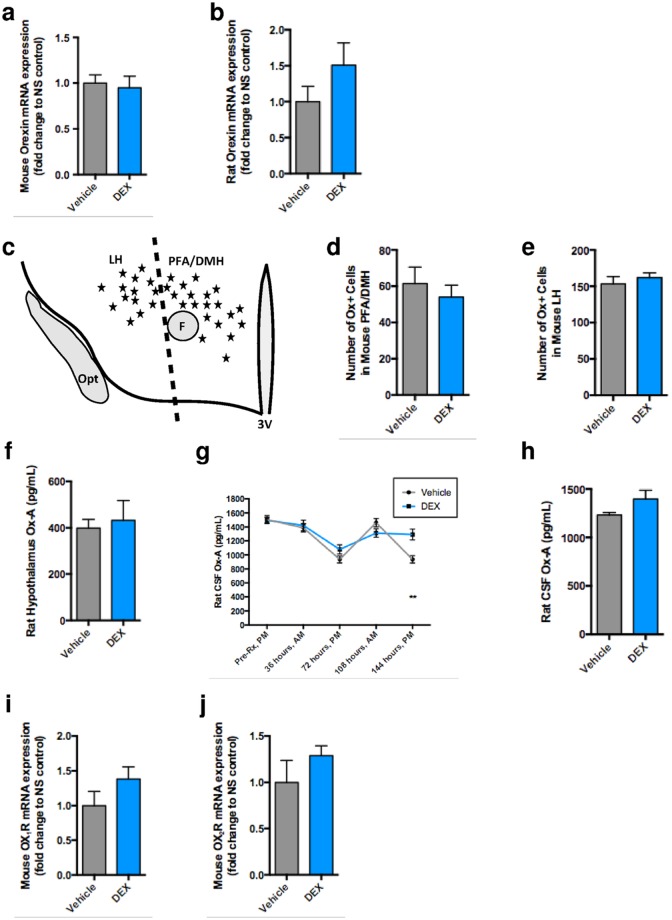
Mice (a, c-e, i-j) and rats (b, f-h) treated with dexamethasone (DEX) versus saline (vehicle) exhibit largely preserved orexin neuron signaling. (a) DEX does not cause a measurable difference in mouse orexin neuron gene expression as measured by RT-PCR (Vehicle *n* = 3, DEX *n* = 5). (b) DEX does not cause a measurable difference in rat orexin neuron gene expression as measured by RT-PCR (Vehicle *n* = 4, DEX *n* = 3). (c) Schematic illustrating the division between LH and PFA/DMH orexin neuron populations. (d, e) DEX does not upregulate mouse orexin neuron gene expression as measured by ISH, as grain clusters per orexin neuron (corresponding to radiolabeled *Hcrt* mRNA) are equivalent in both the DEX and NS animals in both the PFA/DMH and LH (Vehicle *n* = 9, DEX *n* = 8). (f) DEX does not alter total hypothalamic orexin (Ox-A) protein concentration in rats treated with DEX (Vehicle *n* = 7, DEX *n* = 7). (g) A disrupted normal diurnal variation of Ox-A by day 5 of DEX-treatment compared to sham (Vehicle *n* = 8, DEX *n* = 7) **p < 0.01. (h) On repeat testing, CSF Ox-A concentration on day 5 was equivalent between groups (Vehicle *n* = 7, DEX *n* = 7). (i, j) DEX did not cause a measurable difference in mice orexin 1 receptor gene expression (Vehicle *n* = 4, DEX *n* = 3) or orexin 2 receptor gene expression (NS *n* = 4, DEX *n* = 3) as measured by RT-PCR. Data, unless otherwise specified, represent orexin levels at the expected nadir (evening) on day 5 of DEX or sham treatment. Data are expressed as mean ± SEM.

To examine the effect of DEX on orexin physiology, we measured orexin gene expression, neuropeptide production, and receptor expression following DEX treatment compared to control. First, we measured total *prepro-orexin* gene expression at the end of the dark cycle (expected nadir) following a 5-day burst of DEX. We obtained orexin mRNA from DEX-treated mice (*n* = 5) and vehicle-treated mice (*n* = 3) and found comparable mean total orexin mRNA production (DEX, 0.95-fold decrease ± 0.1, p = 0.7)(Fig 1A). We performed the same experiment in rats (DEX, *n* = 3; vehicle, *n* = 4) and similarly did not detect a significant difference in total orexin mRNA between groups (DEX, 1.5-fold increase ± 0.3, p = 0.2) (Fig 1B).

Second, because anatomically distinct subpopulations of orexin neurons have different projections and functions, we examined whether DEX administration increases orexin gene expression at the end of the dark phase in the sleep-regulating neurons in the PFA-DMH compared to the primarily reward-regulating neurons in the LH (Fig 1C). We did not detect a difference in the number of orexin-positive cells in the PFA-DMH in DEX-treated mice (*n* = 8) compared to vehicle-treated mice (*n* = 10) (mean-DEX = 54 ± 7; mean-vehicle = 61 ± 9 cells, *t*(16) = 0.65, *p* = 0.3) or in the LH (mean-DEX = 162 ± 7; mean-vehicle = 153 ± 10 cells, *t*(16) = 0.68, *p* = 0.5)(Fig 1D and 1E).

Third, in order to directly measure orexin neuron output, we measured total orexin protein concentration in the hypothalamus following DEX. We found no difference in total hypothalamic orexin concentration between DEX-treated rats (*n* = 7) and vehicle-treated rats (*n* = 7) (mean-DEX = 432 ± 85.6 pg/mL; mean-vehicle = 399 ± 37.9 pg/mL, *t*(12) = 0.36, *p* = 0.7)(Fig 1F). Fourth, because orexin signaling is clinically measured by CSF orexin concentrations, we measured rat orexin CSF levels at four time points, every 36 hours, during a 5-day course of treatment. Rats treated with DEX (*n* = 5) compared to vehicle (*n* = 7) exhibited disrupted orexin rhythmicity with elevated nadirs, most prominently and significantly at the end of the 5-day course of DEX (mean-DEX = 1,291 ± 77.2 pg/mL; mean-vehicle = 936 ± 51.5 pg/mL, *t*(4) = 0.5, *p* = 0.002)(Fig 1G). To confirm these data, rat orexin concentration in the CSF was measured on day 5 at the expected orexin nadir following DEX (*n* = 7) and vehicle (*n* = 7) treatment; we found no difference in mean orexin concentrations (mean-DEX = 1,398 ± 89.8 pg/mL; mean-vehicle = 1,234 ± 22.6 pg/mL, *t*(12) = 1.8, *p* = 0.1)(Fig 1H).

Finally, we examined the impact of corticosteroids on the orexin receptors of downstream neurons in the hypothalamic area. We measured comparable mean total OX_1_R and OX_2_R gene expression in response to DEX (*n* = 3) and vehicle (*n* = 4) (OX_1_R, DEX, 1.4-fold increase ± 0.2, p = 0.2)(OX_2_R, DEX, 1.3-fold increase ± 0.1, p = 0.4)(Fig 1I and 1J). Thus, we did not find evidence that DEX increases orexin activity by increasing the transcription of the receptor mRNA.

Data on mice and rat body weights, food intake, plasma and CSF glucose levels are shown in S2 Fig. All DEX-treated rodents experienced weight loss, and no vehicle-treated animal experienced weight loss. No animals exhibited hypophagia. Rats were found to have hyperglycemia within 36 hours of initiating DEX treatment, whereas mice were normoglycemic throughout DEX and vehicle treatments.

Taken together, these data indicate that physiologic orexin signaling is preserved following DEX treatment.

### Dexamethasone administered to children undergoing induction therapy for ALL is not associated with an increase in CSF orexin concentration

**Fig 2:**
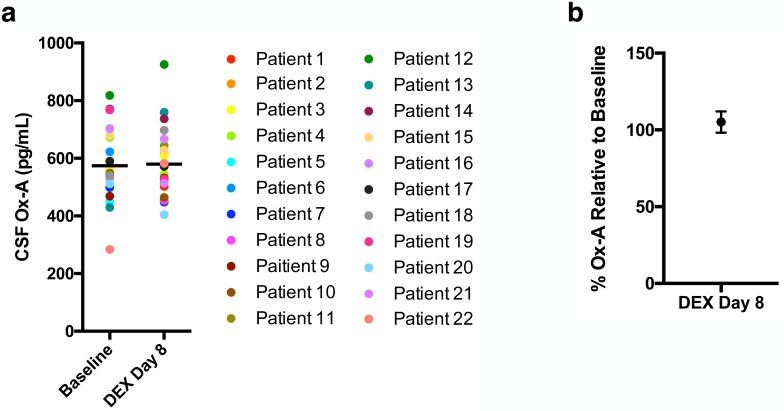
Human CSF orexin levels. (a) Individual patient CSF orexin levels at baseline and then 8 days into dexamethasone (DEX) therapy for ALL. Mean baseline orexin concentration is equivalent to mean DEX day 8 orexin concentration. (b) There was no significant change in mean orexin level from baseline to DEX day 8 (*n* = 22). Data are expressed as mean ± SEM.

To test the hypothesis that corticosteroids upregulate the orexin system, we measured CSF orexin concentrations in children undergoing DEX treatment as part of induction therapy for ALL. We performed orexin measurements on the initial 22 paired samples. None of the children carried a previous diagnosis of sleep abnormalities, and none of the children experienced acute neurological toxicity from chemotherapy. Mean pre-DEX baseline CSF orexin concentrations were 574 ± 26.6 pg/mL compared to 580 ± 126.1 pg/mL at day 8 of DEX (p = 0.8)(Fig 2A). When compared to matched baseline orexin levels, mean increase in CSF orexin level was 5.2 ± 6.9% (p = 0.6)(Fig 2B). These data also suggest that orexin signaling is preserved during DEX administration in children.

## Discussion

In children undergoing therapy for ALL, sleep disturbance is a common and distressing symptom associated with corticosteroid chemotherapy. However, the neural mediators of this adverse reaction are unknown. Evidence that phenotypic patterns of steroid-induced sleep disturbance are strikingly similar to behavioral changes associated with supraphysiologic orexin output led us to hypothesize that dexamethasone causes sleep disruption by altering normal orexin physiology. This study was timely, as the FDA recently approved an orexin receptor antagonist that reduces orexinergic tone as a drug to improve sleep. Based on our experimental system, we demonstrate that orexin signaling is largely preserved during dexamethasone therapy. We show that orexin gene expression, both globally and specifically in the PFA-DMH, the subset of hypothalamic orexin neurons linked to sleep behavior, is unaltered by dexamethasone. We show that orexin neurons produce equivalent levels of orexin protein, found both in the hypothalamus and in the CSF of rodents and in the CSF of humans, following dexamethasone and vehicle therapy. We also show that orexin activity is not increased by increasing the expression of downstream orexin receptors. In these experiments, the response of the orexin system to corticosteroids was studied at both the mRNA and protein level, in rodents and in humans, and the consistency of the data presented here suggests that orexin pathology is not the key mediator of steroid-induced sleep disturbance.

Previous studies have explored various hypothesized mechanisms through which corticosteroids may cause sleep disturbance. Vallance, et al. evaluated the relationship between dexamethasone and sleep and found a correlation between a polymorphism in glucocorticoid metabolism [*AHSG C>G* (Thr238Ser) exon 7 genotype], upregulation of this *ASHG* hepatic protein, and sleep disturbance.[9] The same study evaluated the connection between serum albumin concentration on patients undergoing dexamethasone therapy and sleep and found no association.[9] There may exist a relationship between elevated IL-6 and TNF, two proinflammatory cytokines associated with fatigue and sleep disturbances in patients with chronic illnesses.[39, 40] In studying this relationship in children undergoing steroid chemotherapy for ALL, Vallance, et al. found a correlation between a polymorphism in the *TNF* gene (-308G>A) and steroid-associated sleep disruption.[41] In our study and presented in supporting information, we also investigated the effect of dexamethasone on melanin-concentrating hormone (MCH), an influential regulator of the ascending arousal system that controls the switch from REM sleep to NREM sleep; we found no evidence that dexamethasone affects MCH physiology (S1 Supplemental Methods, S1 Supplemental Results and S3 Fig).

There are several limitations to this study. First, mice and rats receiving dexamethasone invariably exhibited significant weight loss compared to controls, though none were hypophagic. Rats were found to have both hyperglycemia and elevated glucose concentrations in the CSF. Therefore, their weight loss was attributed to steroid-induced hyperglycemia, polyuria, and dehydration. Mice, however, did not develop hyperglycemia; additionally, dexamethasone- and vehicle-treated mice had equivalent total body water, total body fat, and gastrocnemius muscle weight at the end of 5 days of treatment. Therefore, we were not able to explain the dexamethasone-associated weight loss in mice. These findings, most notably the speed and severity of the hyperglycemia and weight loss, were unexpected, because literature suggests that dexamethasone causes an insulin resistance that necessitates longer-term glucocorticoid exposure.[42, 43] Overall, this significant toxicity in rodents is a potential confounder to our data. Furthermore, the use of both mice and rats is another potential weakness of our study. Both animals bring advantages to this type of basic research which prompted our use of both: mice have the advantage of ease of access to relevant genetic models and a very robust body of basic neuroscience research, particularly regarding the role of orexin in arousal and sleep stability, whereas rats are larger, facilitating ease of sampling, and have a robust body of literature supporting them as models of neuroendocrine regulation. In the overwhelming majority of cases, the neurophysiology of mice and rats is similar, and we attempted to capitalize on the strengths of each model to support our research.

Additionally, while we were able to control the time of day of the CSF collection in the rodent models, we were not able to do so for acquisition of the human CSF samples, given practical scheduling constraints. In rats and in squirrel monkeys, orexin levels in the CSF follow a predictable diurnal pattern of peaking near the end of the wake period and while the nadir is during the sleep period.[10, 44] In contrast, human orexin levels peak during sleep in the early morning; however, diurnal cycle-related variability is small, at no more than 10%.[45, 46] Ripley and colleagues also demonstrated the consistency and stability of orexin CSF levels within subjects in the setting of neurologic diseases and malignancies, but highlighted the wide variability between subjects.[47] We were able to capitalize on this intrasubject stability by collecting paired measurements from each patient, enabling the correlation to an internal control. Thus, the timing of CSF collection in humans may have had minimal impact on our ability to detect a change in CSF orexin concentrations after seven days of dexamethasone therapy. Other potential confounders to our human data may include unknown effects on the orexin system of concomitant chemotherapy and supportive care medications, disruption of the normal home environment, and the fatigue associated with cancer *per se*.[48]

Understanding the causal mechanisms of steroid-induced sleep disturbance is important, given the pervasiveness of this corticosteroid toxicity and the potential for intervention. Future research should continue to explore possible mechanisms underlying steroid-induced sleep disturbance.

## Supporting Information

S1 Fig Technique for serial sampling of rat cerebrospinal fluid (CSF). (TIF)

S2 Fig Dexamethasone (DEX) causes significant weight loss mice (a-d) and rats (e-g) as compared to saline (vehicle). (TIF)

S3 Fig Mice (A, C) and rats (B) treated with dexamethasone (DEX) versus saline (vehicle) exhibit preserved MCH function. (TIF)

S1 Supplemental Methods Measuring the impact of corticosteroids on the hypothalamic Melanin Concentrating Hormone (MCH). (DOCX)

S1 Supplemental Results MCH signaling appears to be preserved following dexamethasone administration. (DOCX)
