# Supplementary figures and images for: Dexamethasone Chemotherapy Does Not Disrupt Orexin Signaling
Source: PLoS One. 2016 Dec 20;11(12):e0168731. doi: 10.1371/journal.pone.0168731 (PMC5173249; doi:10.1371/journal.pone.0168731)

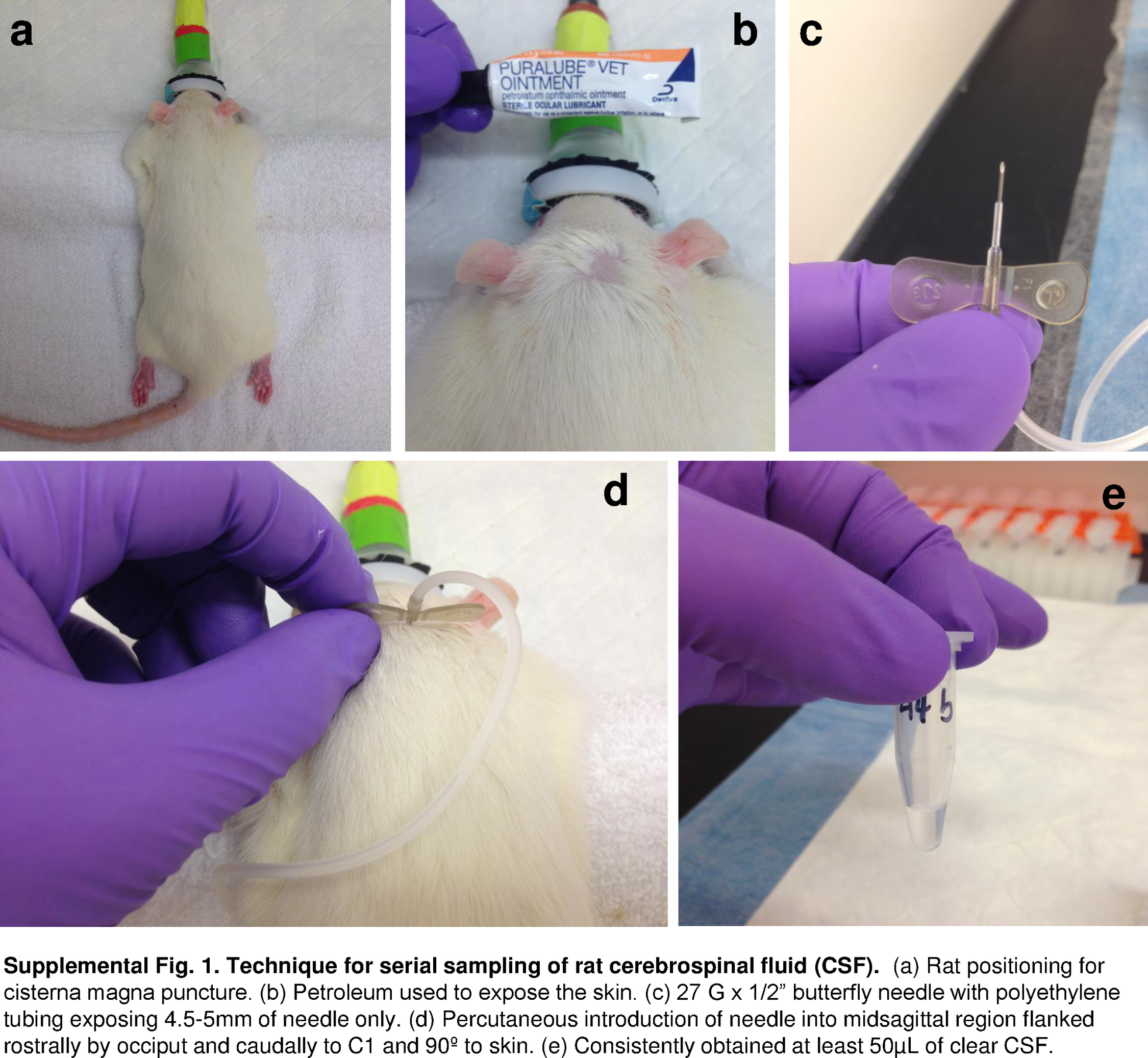

Supplement: S1 Fig — (TIF) [file pone.0168731.s001.tif]

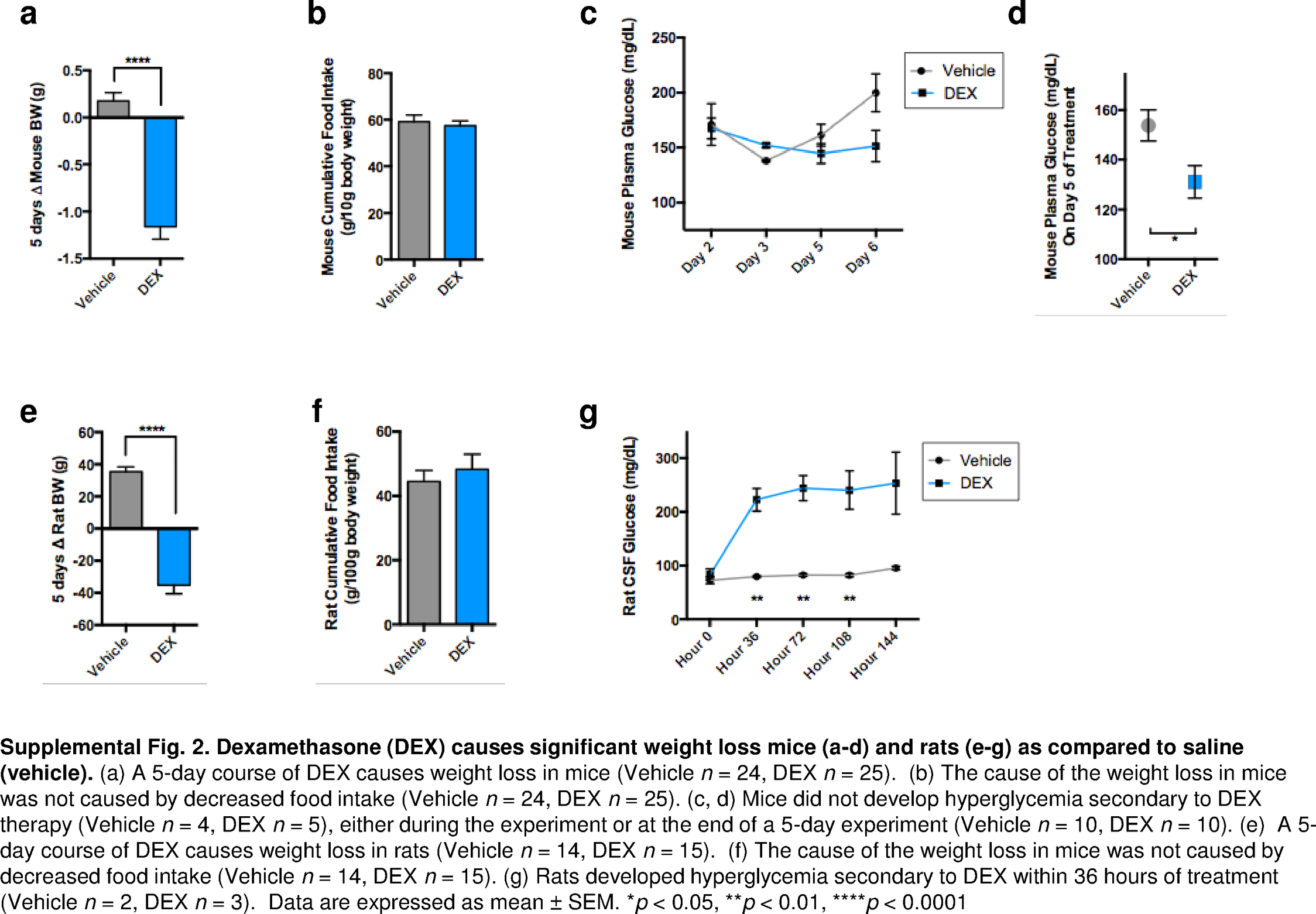

Supplement: S2 Fig — (TIF) [file pone.0168731.s002.tif]

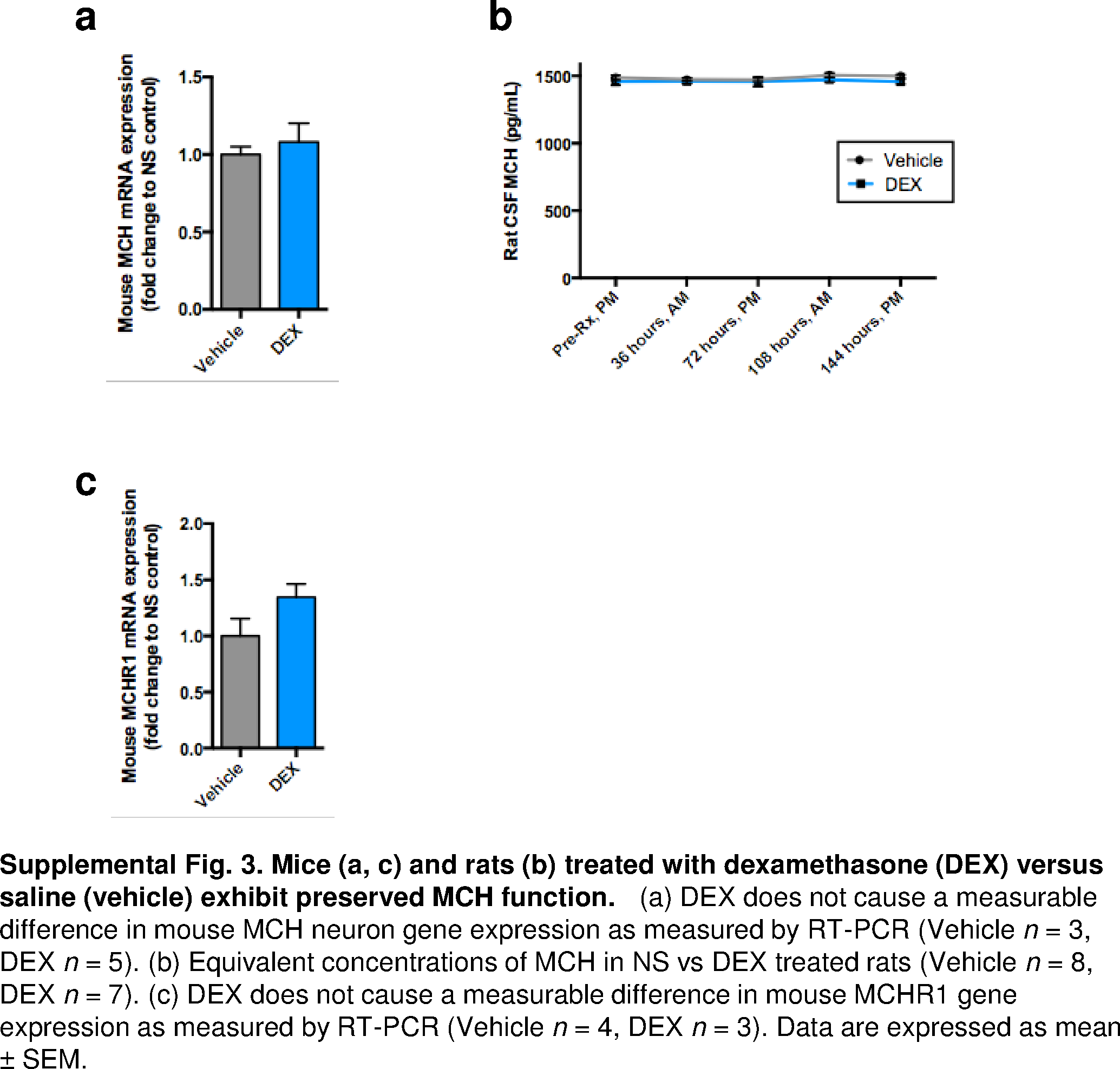

Supplement: S3 Fig — (TIF) [file pone.0168731.s003.tif]
