# Supplementary material for: Dexamethasone Chemotherapy Does Not Disrupt Orexin Signaling
Source: PLoS One. 2016 Dec 20;11(12):e0168731. doi: 10.1371/journal.pone.0168731 (PMC5173249; doi:10.1371/journal.pone.0168731)
Supplement: S1 Supplemental Methods — (DOCX) [file pone.0168731.s004.docx]

**S1 Supplemental Methods**

**Measuring the impact of corticosteroids on the hypothalamic Melanin Concentrating Hormone (MCH)**

Like orexin neurons, MCH neurons are hypothalamic neurons that play a crucial role in the sleep-wake cycle.[1] MCH neurons regulate REM sleep episode duration by controlling the REM to NREM switch, whereas orexin controls the transition between wakefulness and sleep.[2] While our hypothesis focused on the relationship between corticosteroids and the orexin system, we simultaneously investigated the MCH system as well. We measured MCH gene expression, MCH protein production, and MCH receptor gene expression using the same techniques described above for orexin, but using MCH and MCH receptor primer-probes (Applied Biosystems) and a MCH RIA kit (Phoenix Pharmaceuticals, Burlingame, CA).

**Supplemental References**

1. Adamantidis A, de Lecea L. Physiological arousal: a role for hypothalamic systems. Cell Mol Life Sci. 2008;65(10):1475-88. doi: 10.1007/s00018-008-7521-8. PubMed PMID: 18351292.

2. O'Leary LA. Orexin and melanin-concentrating hormone neurons: a hypothalamic interface for sleep and feeding regulation. Bioscience Horizons. 2014;7(0):hzu008-hzu. doi: 10.1093/biohorizons/hzu008.
