# Supplementary material for: Dexamethasone Chemotherapy Does Not Disrupt Orexin Signaling
Source: PLoS One. 2016 Dec 20;11(12):e0168731. doi: 10.1371/journal.pone.0168731 (PMC5173249; doi:10.1371/journal.pone.0168731)
Supplement: S1 Supplemental Results — (DOCX) [file pone.0168731.s005.docx]

**S1 Supplemental Results**

**MCH signaling appears to be preserved following dexamethasone administration.**

We obtained MCH mRNA from DEX-treated mice (*n* = 5) and vehicle -treated mice (*n* = 3) and found comparable mean total MCH mRNA production (DEX, 1.1-fold increase ± 0.1, p = 0.6)(S3 Fig A). We measured CSF MCH protein concentrations at four time points, every 36 hours, during a 5-day course of treatment. Rats treated with DEX (*n* = 7) compared to vehicle (*n* = 8) had serially equivalent MCH levels (p > 0.08 at each time point)( S3 Fig B). Finally, we measured equivalent MCHR1 gene expression in response to DEX (*n* = 3) and vehicle (*n* = 4) (DEX, 1.3-fold increase ± 0.1, p = 0.2)( S3 Fig A). Together, these data indicate the MCH signaling is preserved following DEX treatment.
